# Plantar Heel Pain Management: A Survey of UK Registered Healthcare Professionals

**DOI:** 10.1002/jfa2.70087

**Published:** 2025-10-11

**Authors:** Chris Drake, Lara Chapman, Carole Burnett, Jill Halstead, Anthony Redmond, Edward Roddy, Richard Wilkins, Anne‐Maree Keenan

**Affiliations:** ^1^ Leeds Institute of Rheumatic and Musculoskeletal Medicine University of Leeds Leeds UK; ^2^ Mid Yorkshire Teaching NHS Trust Wakefield UK; ^3^ NIHR Leeds Biomedical Research Centre Leeds UK; ^4^ Research and Innovation Department Leeds Teaching Hospitals NHS Trust Leeds UK; ^5^ Leeds Institute of Medical Research University of Leeds Leeds UK; ^6^ Leeds Community Healthcare NHS Trust Leeds UK; ^7^ Centre for Musculoskeletal Health Research School of Medicine Keele University Keele Staffordshire UK; ^8^ Haywood Academic Rheumatology Centre Midlands Partnership University NHS Foundation Trust Burslem Staffordshire UK; ^9^ Podiatry Department Leeds Teaching Hospitals NHS Trust Leeds UK; ^10^ School of Healthcare University of Leeds Leeds UK

**Keywords:** healthcare professional, management, plantar heel pain, survey, UK

## Abstract

**Background:**

Multiple healthcare professions are involved in the management of plantar heel pain (PHP). Professional diversity can lead to varied practice and treatment choices. Understanding PHP management can aid clinical benchmarking, inform knowledge mobilisation strategies, and may support shared decision making between patients and referrers. This study explored the management of plantar heel pain by United Kingdom (UK) registered healthcare professionals.

**Methods:**

A cross‐sectional, online survey using the Jisc Online Surveys platform. The survey was distributed to UK healthcare practitioners via digital channels, including professional bodies, special interest groups and social media.

**Results:**

Four hundred and six professionals responded, predominantly podiatrists (181; 44.6%) and physiotherapists (144; 36.5%). The remaining 18% comprised orthotists, osteopaths, orthopaedic surgeons, General Practitioners (GPs), nurses, rheumatologists, sport and exercise rehabilitation specialists, and a consultant in sport and exercise medicine. Most respondents (247; 60.8%) did not commonly use imaging to guide PHP management. The majority (359; 88.4%) provided physical interventions: strengthening (88%), stretching (85.5%) and balance (65.2%) were frequently used. Prefabricated orthoses (56.3%) were more frequently used than custom orthoses (24.2%). Treatments employed by podiatrists and physiotherapists were similar. Advice on nonsteroidal anti‐inflammatory drugs (NSAIDs) (226; 83%), and simple analgesics (e.g., paracetamol) (221; 81%) to manage pain was commonly provided. Only 6.4% frequently performed steroid injections. Patient information was commonly provided (359; 88%); however, most practitioners (297; 83%) lacked resources in languages other than English.

**Conclusion:**

Healthcare professionals in the UK favour exercise and prefabricated orthoses when managing PHP.

## 1 Introduction

Plantar heel pain (PHP) is broadly characterised by pain under the heel, which is often worse when people take their first steps in the morning, and after longer periods of weight bearing [1]. Around 1 in 10 people aged over 50 will experience PHP: most will report disabling levels of pain [2], with around half of people with PHP reporting persistent symptoms at 10 years [3]. People with PHP often struggle to remain physically active [4, 5], experience weight gain [2] and report a negative impact on health‐related quality of life [5], including reduced social participation and isolation [4]. Often, they have unmet needs and expectations, with doubts about the cause, management and prognosis of their symptoms [6].

Accessing treatments is likely to be more beneficial than a watchful waiting approach for people with PHP; however, no single treatment demonstrates superiority over others [7]. Clinical guidelines have sought to strengthen recommendations through a mixed method approach [8]; however, the underpinning quality of primary studies is often low, and impaired by small sample sizes. The myriad of treatment options is further complicated by the number of professions that utilise them.

In the United Kingdom (UK), PHP poses a considerable burden to healthcare services [9, 10, 11, 12, 13]. Referrals are made to various health professions [2], including doctors, nurses, podiatrists, physiotherapists, orthotists and osteopaths, with patients seen in both primary and secondary care settings. Additionally, nonmedical practitioners are increasingly working in extended roles, such as advanced clinical practitioners (ACP), advanced specialists in musculoskeletal (MSK) care, and first contact practitioners (FCP) in primary care [14, 15], and will also encounter PHP. Physiotherapists and podiatrists may provide physical interventions for PHP, such as exercise and orthoses [16, 17, 18], yet it is unclear if current practice varies between these and other professions. Variation between professional clinical practice has the potential to create inequality [19], in a healthcare system that is already complex and under significant care delivery challenges.

Understanding current practice is a key component of clinical benchmarking [20]. As evidence on best practice evolves, patterns identified in practice can also inform knowledge mobilisation strategies. Additionally, gaining insight into the treatments provided by different health professions may support shared decision‐making between patients and referrers [21]; enabling patients to make informed, personalised decisions about their care [22].

There is a clear need to understand how different professions manage PHP in the UK. In response to this, we surveyed UK registered healthcare professionals who are involved in managing PHP. The survey aimed to describe how PHP is managed by different healthcare professions. The secondary aims were to explore variation in service provision and barriers to treatment.

## 2 Methods

This study utilised a cross‐sectional, online survey to explore the management of PHP in the UK using the Jisc Online Surveys platform [23]. Participants were required to acknowledge they had read and understood the participant information sheet, and consented to the proposed data use, before proceeding with the survey. The survey was accessible for six weeks between May 2024 and June 2024. Ethical approval was provided by the University of Leeds, School of Medicine Ethics Committee (MREC‐23‐017).

## 3 Questionnaire Design

The survey questions were informed by the literature and developed iteratively by the research team, experienced clinicians and academics in the field of foot pain. These questions were piloted with clinicians from a variety of professional backgrounds, including physiotherapy, podiatry, rheumatology, first contact practitioners and orthopaedic surgery. The final survey (Supporting Information S1) included 31 questions, broadly categorised into participant demographics, referral pathways, imaging and clinical features, management practices, outcomes measures, service provision and barriers to treatment. The survey is reported in accordance with the Consensus‐based checklist for Reporting of Survey Studies (CROSS) reporting guideline [24].

## 4 Participants

UK registered healthcare professionals involved in the management of PHP were eligible to complete the online survey. The survey was distributed through various digital channels, including professional bodies, clinical interest groups (Supporting Information S2) and disseminated through social media platforms such as X and Facebook. There is limited guidance on sample size for surveys of practice. No limit was set on sample size, and the distribution methods were designed to maximise responses. Responses from previous studies that have surveyed PHP practice in the UK ranged from 124 to 256 [16, 17, 18].

## 5 Data Analysis

A descriptive analysis of survey data was conducted using SPSS v29 (Armonk, NY: IBM Corp). The categorical survey data were analysed using descriptive statistics (frequency/percentages). Free text comments were categorised and then analysed descriptively (Supporting Information S3). These statistics are presented in frequency tables.

## 6 Results

### 6.1 Demographics

Four hundred and six respondents from 10 healthcare professions completed the survey (Table 1). Most were based in England (371; 91.4%), and over half worked in the National Health Service (NHS) (240; 59.1%). The largest number of responses were from podiatrists (181; 44.6%) and physiotherapists (148; 36.5%). Responses were also received from orthotists (30; 7.4%), osteopaths (25; 6.2%), orthopaedic surgeons (9; 2.2%), general practitioners (GP) (5; 1.2%), nurses (3; 0.7%), rheumatologists (2; 0.5%), sport and exercise rehabilitation specialists (2; 0.5%) and a consultant in sport and exercise medicine (1; 0.25%). Respondents principally working in extended roles: advanced clinical practitioners (97; 23.9%), advanced specialists in musculoskeletal care (92; 22.7%) and first contact practitioners in primary care (51; 12.6%).

**TABLE 1:** Table presenting the professional characteristics of the survey respondents.

	Frequency^a^ (%)
Profession	
Podiatrist	181 (44.6)
Physiotherapist	148 (36.5)
Orthotist	30 (7.4)
Osteopaths	25 (6.2)
Orthopaedic surgeon	9 (2.2)
General practitioners	5 (1.2)
Nurse	3 (0.7)
Rheumatologist	2 (0.5)
Sport and exercise rehabilitation specialist	2 (0.5)
Consultant in sport and exercise medicine	1 (0.25)
Extended roles	
Advanced clinical practitioner	99 (24)
Musculoskeletal service practitioner	92 (23)
First contact practitioner	51 (13)
None of the above	164 (40)
Length of practice	
0–5 years	41 (10)
6–10 years	59 (15)
11–15 years	63 (16)
16–20 years	51 (13)
More than 20 years	192 (47)
Principal clinical sector	
NHS	240 (59)
Private practice	110 (27)
Independent sector	8 (2)
I work across sectors	48 (12)
Specific area	
Private practice	114 (28)
Primary care	98 (24)
Secondary care	84 (21)
Community health	56 (14)
Tertiary care	11 (3)
I work across areas	43 (11)
Region	
England	371 (91)
Northern Ireland	6 (1.5)
Scotland	20 (5)
Wales	7 (2)
I work across regions	2 (0.5)

^a^ *n* = 406.

### 6.2 Referrals

**FIGURE 1:**
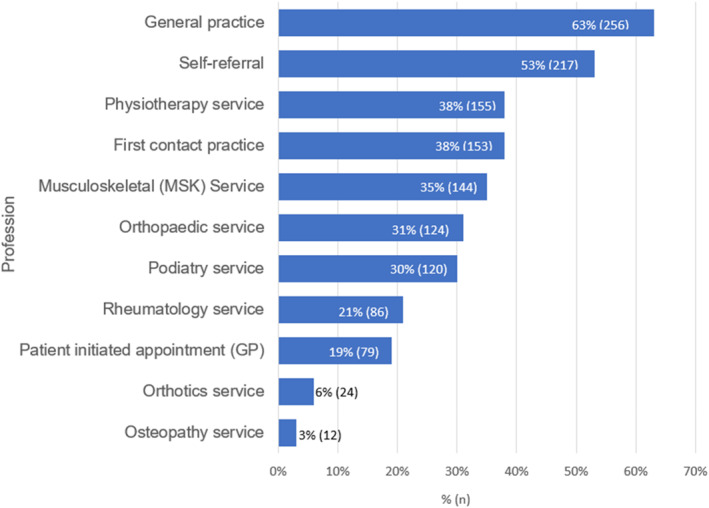
Bar chart showing sources that respondents generally received their PHP referrals from.

**FIGURE 2:**
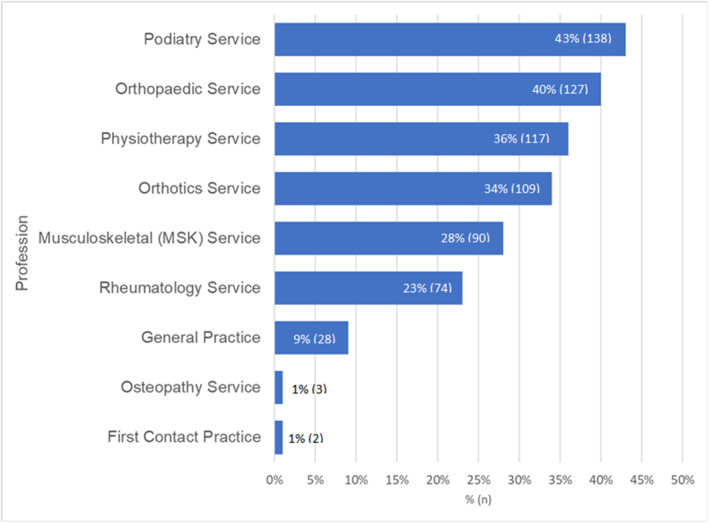
Bar chart showing professions and services that respondents make onward referrals for PHP.

Referrals for PHP were received from a wide range of professions (Figure 1). GP referral was the most selected referral source (256; 63.1%), followed by self‐referral (217; 53.4%). Over three‐quarters of respondents reported making onward referrals to other professions (321/406), most commonly to podiatry (138; 43%) and orthopaedic services (127; 40%, Figure 2).

### 6.3 Imaging

Most respondents reported never or rarely using diagnostic imaging to guide management (247; 60.8%), with a quarter sometimes using it (111; 27.3%). Only a small proportion of respondents reported frequently or very frequently using imaging (48; 11.8%), over half of whom were advanced clinical practitioners (28; 6.9%).

**FIGURE 3:**
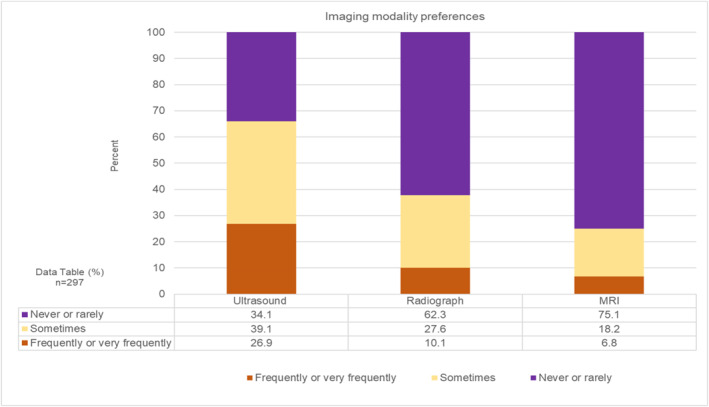
Stacked bar chart showing imaging modality preferences.

Of 297 respondents who used imaging to guide management at any frequency (see Figure 3): ultrasound was more frequently or very frequently used (80; 26.9%), compared to plain radiographs (30; 10.1%) and MRI (20; 6.8%).

**FIGURE 4:**
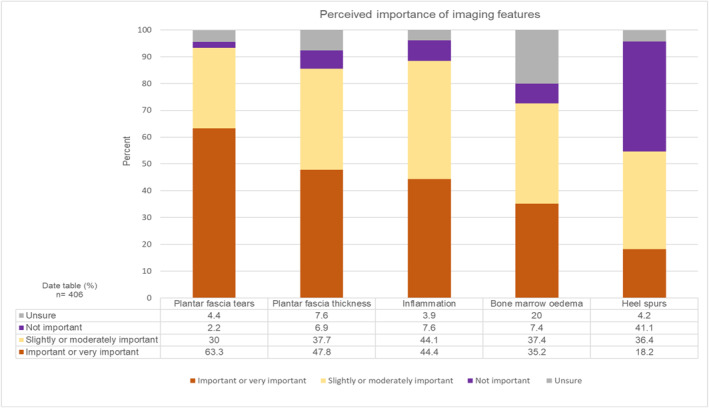
Stacked bar chart showing how respondents perceived the importance of imaging features.

In terms of which imaging features were considered important (Figure 4), plantar fascia tears were most reported as important or very important (257; 63.3%), followed by plantar fascia thickness (194; 47.8%), and inflammation (180; 44.4%). Heel spurs were most frequently reported as not important (167; 41.1%), and bone marrow oedema was the feature which most people were unsure of (81; 20%).

Free text responses identified other imaging features perceived to be important (see Supporting Information S3): bone features (11; 2.7%), plantar fat pad features (10; 2.5%), rheumatological features (7; 1.7%), muscle features (6; 1.5%), other plantar fascia features (5; 1.2%), neural features (3; 0.7%) and tumour (3; 0.7%).

### 6.4 Clinical Features

**FIGURE 5:**
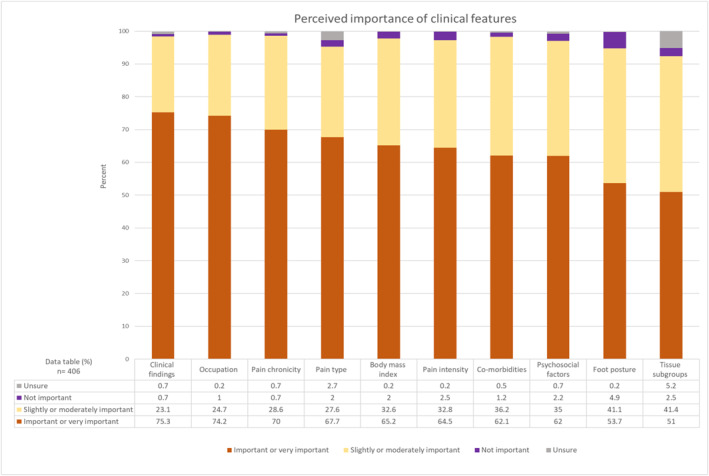
Stacked bar chart showing how respondents perceived the importance of clinical features.

Clinical assessment findings (e.g., muscle tightness, strength, and range of motion) (306; 75.3%), and occupation (301; 74.2%), were the features most reported as important or very important in their clinical management of PHP. Tissue subgroups (e.g., fasciopathy, inflammation, bone marrow oedema) (207; 51%) were the least frequently reported as important or very important and were the feature that most people were unsure about (21; 5.2%) (Figure 5).

Other clinical features perceived to be important in the free text included: footwear (19; 4.7%), activity levels (16; 3.9%), biomechanics (12; 3%), trauma (9; 2.2%), patient demographics (6; 1.5%) and hormonal influences (3; 0.7%).

### 6.5 Injections

Referring onwards to others to perform steroid injections for PHP was not common practice. Only 20 (4.9%) respondents reported frequently or very frequently referring onwards for injections, 115 (28.1%) sometimes and 271 (66.8%) never or rarely referring for injections. Of the 20 respondents who frequently or very frequently referred for injections, 14 (70%) worked in an extended role that is ACP, FCP or MSK.

Over three‐quarters of respondents reported never or rarely personally performing steroid injections for PHP (331; 81.5%). A small (49; 12.1%) proportion sometimes performed them; however, few (26; 6.4%) performed them frequently or very frequently. Of the respondents who frequently or very frequently performed injections for PHP, 21 (81%) worked in an extended role.

### 6.6 Medications

Most respondents reported that they provided medication advice to patients to manage pain (272; 67%). Non‐steroidal anti‐inflammatory drugs (NSAIDs) (226; 83%) and simple analgesics (221; 81%) were similarly advised. Other medication advice included in the free text: neuropathic pain medications (7; 2.6%), co‐codamol (2; 0.7%), disease‐modifying anti‐rheumatic drugs (2; 0.7%) and corticosteroids (1; 0.4%).

### 6.7 Physical Interventions

**FIGURE 6:**
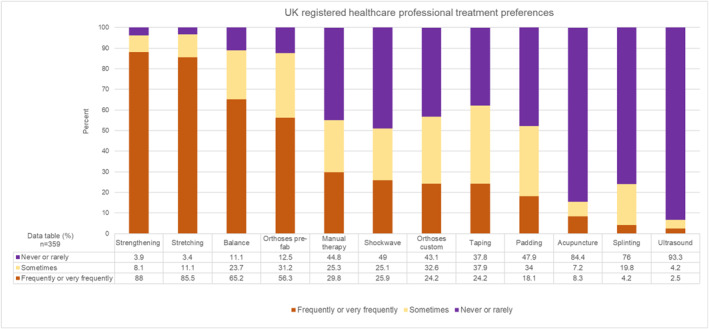
Stacked bar chart showing UK registered healthcare professional treatment preferences.

Most respondents provided physical interventions for PHP (359; 88.4%). Of 359 respondents, exercise‐based interventions were more commonly used than other interventions, with the most frequently/very frequently provided interventions being strengthening (316; 88%) and stretching (307; 85.5%) exercises (Figure 6). Prefabricated orthoses were also frequently/very frequently used (202; 56.3%), and more so than custom orthoses (87; 24.2%). Most respondents never or rarely used therapeutic ultrasound (335; 93.3%) and acupuncture (303; 84.4%).

**FIGURE 7:**
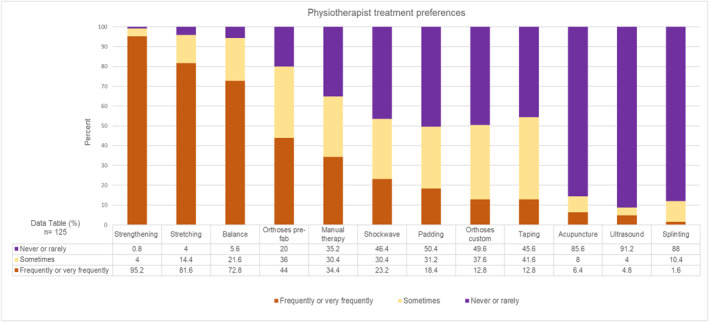
Stacked bar charts showing physiotherapist treatment preferences.

**FIGURE 8:**
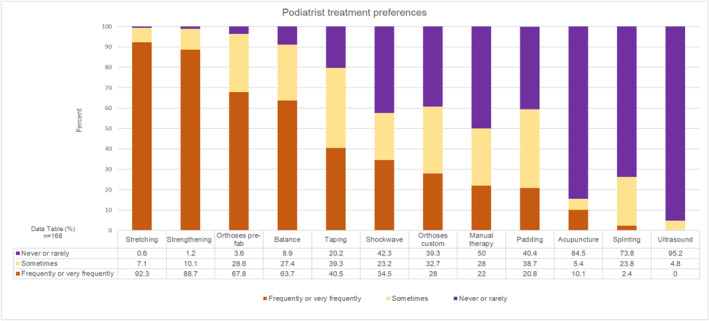
Stacked bar charts showing podiatrist treatment preferences.

To explore differences in physical intervention preferences by profession, analyses of treatments used by physiotherapists (*n* = 125, Figure 7) and podiatrists (*n* = 168, Figure 8) were conducted. These results reflected the main analysis: a predominantly exercise‐based approach to care, with use of prefabricated orthoses.

**FIGURE 9:**
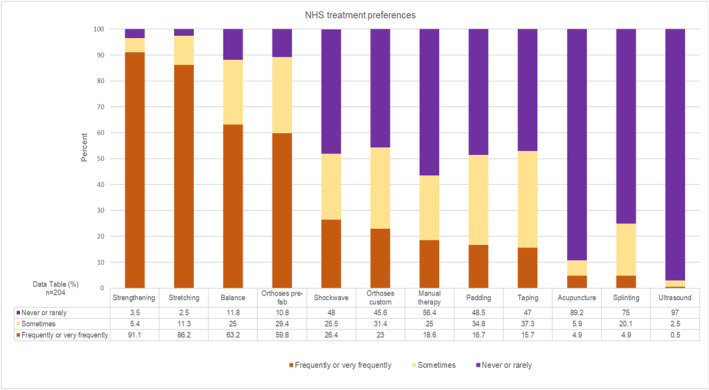
Stacked bar chart showing treatment preferences for those respondents who worked primarily in the NHS.

**FIGURE 10:**
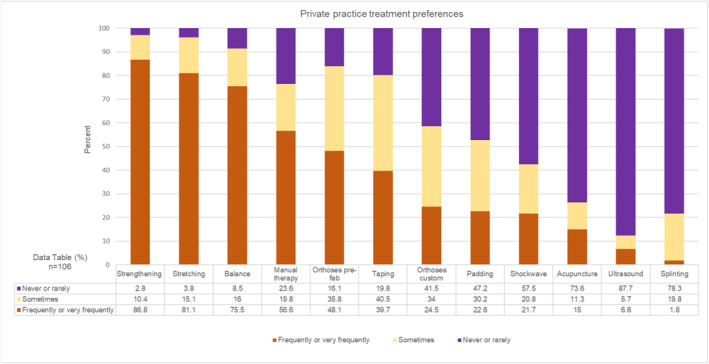
Stacked bar chart showing treatment preferences for those respondents who worked primarily in private practice.

Of the respondents providing physical interventions, treatment preferences were compared between those working principally in the NHS (*n* = 204, Figure 9) and those principally in private practice (*n* = 106, Figure 10). Strengthening, stretching and balance, were the most frequently/very frequently provided interventions, for both clinical sectors. Manual therapy was more frequently/very frequently provided in private practice (60; 56.6%), than prefabricated orthoses (51; 48.1%).

### 6.8 Advice

**FIGURE 11:**
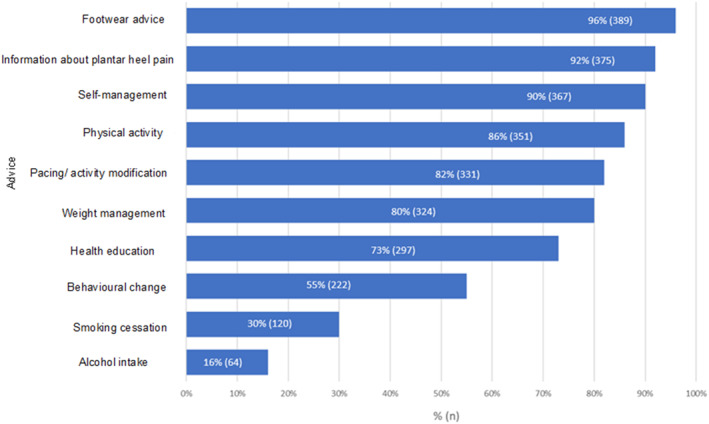
Bar chart representing the type of advice provided to people with PHP.

Respondents routinely provided a wide range of nonpharmacological advice to patients (see Figure 11). The most routinely provided advice concerned footwear (389; 96%), information about PHP (375; 92%) and self‐management advice (367; 90%). The least routinely provided advice was regarding smoking cessation (120; 30%) and alcohol intake (64; 16%).

### 6.9 Sources Informing Management of Plantar Heel Pain

**FIGURE 12:**
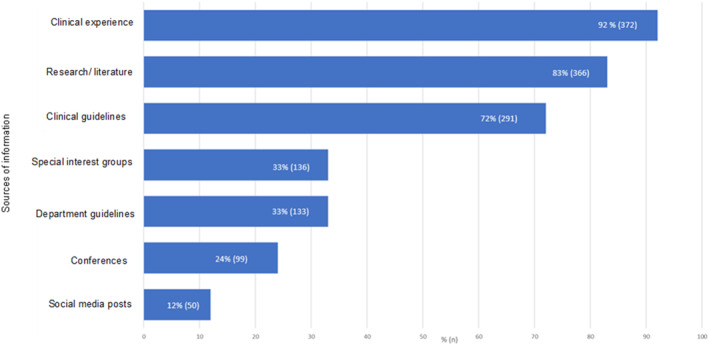
Bar chart representing the sources of information that are important in informing respondents PHP management.

Over 90% of respondents (372; 92%) reported that clinical experience was one of the most important sources in informing management of PHP. Research/literature (336; 83%), and published guidelines (291; 72%) were also frequently reported as important sources. Social media posts (50; 12%) were the least frequently reported as important sources of information (Figure 12).

### 6.10 Patient Information

Most respondents provided patient information as part of their management (359; 88%). Paper information was most routinely provided (238; 66%), followed by webpage (183; 51%) and app based (130; 36%). Of those who provided patient information, over three‐quarters (297; 83%) reported that they did not have access to resources in languages other than English.

### 6.11 Outcomes

A range of outcomes were felt to be important by respondents. Quality of life was most selected as important (370; 91.1%), followed by pain (352; 86.7%), activities of daily living (351; 86.5%) and function (331; 81.5%). The least commonly selected outcome was adherence (189; 46.6%).

Around half (198; 48.8%) of respondents reported frequently or very frequently measuring composite patient reported outcomes (PROs). An equal proportion reported never or rarely (102; 25.1%) or sometimes (106; 26.1%) measuring them.

Foot‐specific outcome measures were not frequently used, with only 68 respondents (16.7%) using them to inform PHP management. Of those, the Manchester‐Oxford Foot Questionnaire (MOXFQ) was the most frequently used tool (40; 59%) (see Table 2). Generic outcome measurement tools were also used sparingly, with only 75 respondents (18.5%) using them. The EQ5D was the most selected generic measurement tool (50; 67%) (see Table 3). Other generic outcome measures mentioned in free text comments: MSK‐HQ (13; 17.3%), PSFS (3; 4%) and TOMS (2; 2.7%).

**TABLE 2:** Table presenting the use and type of foot specific outcome measures.

Foot specific outcome measurement tools	Frequency^a^	Percent
Manchester‐Oxford foot questionnaire (MOXFQ)	40	59
Foot posture index (FPI)	15	22
Foot and ankle disability index (FADI)	12	18
Foot function index (FFI)	12	18
Foot and ankle outcome score (FAOS)	5	7
Foot and ankle ability measure (FAAM)	4	6
American orthopaedic foot and ankle society (AOFAS) clinical rating scale	3	4
Foot health status questionnaire (FHSQ)	3	4
Self‐reported foot and ankle score (SEFAS)	1	1
Manchester foot pain and disability index (MFPDI)	0	0

^a^ *n* = 68.

**TABLE 3:** Table presenting the use of generic outcome measures.

Generic outcome measurement tools	Frequency^a^	Percent
EQ5D	50	67
Short Form–36 (SF‐36)	9	12
Roles and Maudsley score	0	0

^a^ *n* = 75.

Most respondents used pain scales to inform their practice (323; 79.6%). The most selected pain scale was the numeric pain rating scale (i.e., 0–10) (223; 69%), followed by the visual analogue scale (i.e., 0–100 mm) (124; 38%), and verbal rating scale (i.e., no pain, mild, moderate, severe) (60; 19%). Average pain was the most selected pain intensity measurement (276; 85%), followed by first step pain (157; 49%), and night pain (73; 23%). In free text comments, pain at worst or best was a recurring category (31; 9.6%).

### 6.12 Service Provision

The most common number of treatment sessions provided for people with PHP was 3–4 (163; 40.1%). A smaller proportion provided 1–2 sessions (131; 32.3%), and 5–6 sessions (8; 24.1%). It was uncommon for respondents to provide 7 or more sessions (14; 3.4%).

Respondents generally did not have limitations on the number of treatment sessions they could provide (326; 80.6%). Of the 80 respondents who did have limits, service/commissioning agreements were the most selected reason (50; 63%), followed by waiting list pressures (44; 55%) and staffing pressures (29; 36%).

Around half of respondents had a waiting list for people with PHP to be seen (193; 47.5%). Most of those respondents worked primarily in the NHS (150; 77.8%). Almost 70% (135) reported their waiting list was within 4 months, 22% (54) between 4 and 8 months, and 8% (16) reported their waiting list to be 8 months or over.

High level of referrals was the most selected reason for a waiting list (159; 82%), followed by staffing pressures (133; 69%). Covid‐related backlog was a factor for around a quarter of respondents with a waiting list (52; 27%).

## 7 Discussion

This survey shows GPs are the most common source of referrals, followed by self‐referrals. The high burden of PHP on GP services in the UK is well recognised [2], however, this study suggests there may be a sizeable group of people with PHP who are referred to orthopaedics (Figure 2). Orthopaedic services are known to be under significant pressure with long waiting lists post‐Covid‐19 pandemic [25]; additional burden from PHP referrals is likely to hinder recovery plans.

The use of medical imaging to guide management was not common practice. Ultrasound was the most frequently used imaging modality and may reflect the imaging features respondents perceived were important: plantar fascia thickness and tears, which can be viewed on ultrasound [26], rather than bone marrow oedema which can only be viewed on MRI.

Mechanical factors such as strength, range of motion, and occupation were most frequently reported as important in respondents' clinical management of PHP. Whilst the association of these factors with PHP have been debated [27], recent evidence shows that people with PHP spend more time standing [28], and have reduced foot muscle strength and function [29], compared to those without PHP. The survey did not ask how the presence of features influenced management; however, the wide range of clinical features regarded as important indicates that healthcare professionals are faced with significant clinical complexity when considering interventions.

An exercise‐based approach to management was strongly favoured (strengthening, stretching, and balance), with prefabricated orthoses also commonly used (Figure 6) There is an abundance of physical intervention trials for PHP; however, ambiguity remains in terms comparative effectiveness [7, 8]. A network meta‐analysis found equivocal evidence on the comparative effectiveness of PHP interventions [7]. Sham and placebo were least likely to be effective, with exercise only appearing effective for long term pain and function. The study reported significant variation in the size and precision of effect estimates, which limited conclusions. In contrast, a recent best‐practice guideline recommended plantar fascia stretching, education, and taping, escalating to consideration of shockwave therapy and custom orthoses for sub‐optimal responders [8]. Advice on footwear, information about PHP, and self‐management was commonly provided by respondents in this study, however; only a small proportion frequently used taping, or custom orthoses, demonstrating a level of divergence from those recommendations. Notably, this survey suggests that physiotherapists may now provide more shockwave than previously recognised [16].

Advice on NSAIDs was commonly provided, despite current evidence suggesting that it is one of the least effective treatment options for PHP [7]. NSAIDs are also known to carry a significant risk profile [30]. This is relevant not only to doctors, but also to other health professionals in the UK—such as physiotherapists and podiatrists (where relevant to the foot)—who provide medication advice [31, 32].Corticosteroid injections are not more effective at reducing pain and function than placebo [33], however they were frequently utilised by a small group of mainly extended scope practitioners. This may reflect the complex and undifferentiated patient groups seen in extended scope clinics (i.e., nonresponsive PHP) [34].

Patient‐reported outcomes (PROs) were frequently/very frequently used by around half of respondents; however, foot specific and composite generic outcome measures were used sparingly. The reasons for this are likely multi‐factorial: dozens of foot and ankle PROs exist [35, 36], complicating the choice of tool. Survey length, time burden and health literacy are also factors which can limit outcome collection [37].

Around half of respondents had a waiting list to see people with PHP. The impact of waiting lists on outcomes is unclear, but there is evidence to suggest longer waiting may have detrimental effects on pain, disability, quality of life and psychological symptoms in people with musculoskeletal pain [38], and that shorter waiting times may improve health outcomes [39].

### 7.1 Limitations

There are limitations to this survey, primarily the lack of a formal sampling frame for practitioners who manage PHP. There were low numbers of responses from GPs, nurses, orthopaedic surgeons, and rheumatologists, and without a clear denominator population, the extent of nonresponse and related bias is undetermined. Additionally, there is the possibility of recall bias in asking respondents to reflect on their practice, rather than reviewing clinical encounters. Positively, both podiatrists and physiotherapists were well represented, and this reflects the largest proportion of PHP referrals. The high response rate from these professions is likely to provide valuable insights into their practice. Furthermore, this was the first multi‐professional PHP survey where orthotists and osteopaths were represented, providing a foundation for engagement with those professions in future research.

### 7.2 Future Direction

This survey has implications for future research. In clinical practice, there is some divergence from recent best practice guidance on core treatments [8], such as taping and custom orthoses. However, the comparative effectiveness of these treatments is yet to be determined in large clinical trials. Effect estimates in systematic reviews [7, 8], are generally compromised by small sample sizes and the low quality of primary research. Well‐designed, large‐scale randomised controlled trials (RCTs) are therefore essential to evaluate the comparative effectiveness of PHP interventions. Without such studies, it is challenging to make robust recommendations about treatments. Additionally, mechanism of action studies are needed to explore how treatments work and whether they have varied effects in different individuals.

Establishing a core outcome set for PHP, such as that being developed by the ongoing OMERACT initiative [40], will be critical in developing and selecting PROs that are meaningful to patients, and useable in clinical practice. Respondents commonly provided patient information resources, however over 80% did not have access to languages other than English. This raises concern about the accessibility of patient information for people with PHP; there should be a focus on developing patient resources which are accessible to a wider range of communities.

## 8 Conclusion

UK registered healthcare professionals favour exercise and prefabricated orthoses in their management of PHP. Advice on NSAIDs is commonly provided, despite current evidence suggesting that it is one of the least effective treatment options for PHP. Most clinicians do not have access to patient information resources in languages other than English.

## Author Contributions

**Chris Drake:** conceptualization, methodology, formal analysis, writing – original draft, writing – review and editing. **Lara Chapman:** methodology, formal analysis, writing – original draft, writing – review and editing. **Carole Burnett:** writing – original draft, writing – review and editing. **Jill Halstead:** methodology, writing – original draft, writing – review and editing. **Anthony Redmond:** conceptualization, methodology, writing – original draft, writing – review and editing. **Edward Roddy:** conceptualization, methodology, writing – original draft, writing – review and editing. **Richard Wilkins:** methodology, writing – original draft, writing – review and editing. **Anne‐Maree Keenan:** conceptualization, methodology, formal analysis, writing– original draft, writing – review and editing.

## Ethics Statement

Ethical approval received from University of Leeds, School of Medicine Ethics Committee (MREC 23‐017).

## Consent

Participants were required to provide consent before proceeding with the survey.

## Conflicts of Interest

The authors declare no conflicts of interest.

## Supporting information


Supporting Information S1

Supporting Information S2

Supporting Information S3

## Data Availability

The datasets analysed in this study are available from the corresponding author on reasonable request. Data will be also stored in a repository at the University of Leeds.
