# Supplementary material for: Plantar Heel Pain Management: A Survey of UK Registered Healthcare Professionals
Source: J Foot Ankle Res. 2025 Oct 11;18(4):e70087. doi: 10.1002/jfa2.70087 (PMC12515048; doi:10.1002/jfa2.70087)
Supplement: Supplementary file 1 — Supporting Information S1 [file JFA2-18-e70087-s001.pdf]

A multi-professional survey of the management of plantar heel pain in the United Kingdom.

Participant Information

Purpose

This survey will provide an overview of how different health professions manage plantar heel pain (PHP). It is the first survey open to all the United Kingdom (UK) registered health professions who encounter PHP in their clinical practice.

Why am I being invited to take part?

We are inviting you to complete the survey to help us understand how different health professions manage PHP. It will explore if there is variation in management and service provision between professions. This information will be important for healthcare services when benchmarking and commissioning PHP services. It will also be useful for researchers when planning how best to share the results of studies.

Who can take part in the survey?

If you are a registered health professional in the UK and encounter PHP in your practice, we would love to hear from you. It doesn't matter if you are only involved in certain parts of assessment, treatment, or referral; your views are important to us.

Who is conducting this survey?

The survey is being conducted by a small team of researchers from the Leeds Institute of Rheumatic and Musculoskeletal Medicine (LIRMM), at the University of Leeds. The Chief Investigator, Chris Drake is a PhD candidate at the University of Leeds and this forms part of Chris's HEE/NIHR Clinical Doctoral Research Fellowship (NIHR302199). Ethical approval has been obtained from the University of Leeds, Research Ethics Committee (MREC 23-017.)

What will taking part mean for me?

- Your participation is completely voluntary.
- You can exit the survey at any time before submission.
- There are no foreseeable risks associated with your involvement.
- You will not be asked any personal questions that could identify you.
- For most people, the survey will take no longer than 15 minutes to complete.

What will happen to the information I provide in the survey?

All the information collected is completely anonymous. The anonymous data will be stored at the University of Leeds in a secure location. Please see the university research participant privacy notice: https://dataprotection.leeds.ac.uk/research-participant-privacy-notice/. Whilst you may exit the survey at any time, once your survey has been submitted you will be unable to withdraw it. This is because your submission is anonymous, and data are therefore unidentifiable.

The study results will be presented at conferences and published in open-access peer-reviewed journals. Any outcomes from this study will be announced through the same media channels as the survey. Finally, the study data will be uploaded to the University of Leeds Research Repository to allow open access for researchers and the public.

Thank you for taking your time to complete this survey. We really value all your responses.

Ethical approval has been granted from: *School of Medicine Research Ethics Committee: reference number MREC 23-017*.

If you have any further questions about the survey, please contact:

Study Chief Investigator, Chris Drake, Leeds Institute of Rheumatic and Musculoskeletal Medicine, University of Leeds. Email: c.j.drake@leeds.ac.uk

Primary Supervisor, Professor Anne-Maree Keenan, Faculty of Medicine and Health, University of Leeds. Email: a.keenan@leeds.ac.uk

Survey Questions

Consent Page

To continue with this survey please can you confirm:

1. I am a United Kingdom (UK) registered health professional who encounters PHP in my clinical practice.
2. I have read and understood the participant information and that by continuing with this survey I am providing informed consent for my anonymous data to be stored at the University of Leeds. I understand that results will be presented at conferences and published in open-access peer-reviewed journals, and that the anonymised study data will be uploaded to the University of Leeds Research Repository to allow open access for researchers and the public.

Section 1

Demographics

3. **What is your registered health profession?**

List (choose one)

General Practitioner

Nurse

Orthopaedic Surgeon

Orthotist

Osteopath

Physiotherapist

Podiatrist

Rheumatologist

Other

If you selected Other, please specify: *'Please do not enter any information that may identify you or anyone else in the box'.*

4. In addition to your registered profession would you principally see people with PHP in one of these roles? (select one that applies).

Advanced Clinical Practitioner

First Contact Practitioner (FCP)

Musculoskeletal Service Practitioner (i.e. primary care MSK assessment and triage)

None of the above

5. **How long have you been a practicing healthcare professional?**

List (choose one)

0-5 years

6-10 years

11-15 years

16-20 years

More than 20 years

6. **What clinical sector do you principally work in?**

(Select)

NHS

Independent sector

Private practice

I work across sectors

If you work across clinical sectors please select?

(Select)

NHS

Independent sector

Private practice

Which specific area

Primary care

Secondary care

Community health

Tertiary care

Private practice

I work across areas (please select)

(Select options across areas)

7. **Which region in the UK do you work in?**

List (choose one)

England

Northern Ireland

Scotland

Wales

Across regions (Free text)

Section 2

8. **Where do you generally receive referrals for PHP from?**
   Please select all that apply

First Contact Practice

General Practice

Musculoskeletal (MSK) Service

Orthopaedic Service

Orthotics Service

Osteopathy Service

Patient initiated appointment (General Practice)

Physiotherapy Service

Podiatry Service

Rheumatology Service

Self-referral

Other (free text) 'Please do not enter any information that may identify you or anyone else in the box'.

9. **How long would people with PHP generally have had symptoms when they are seen in your service?**

<3 months

3-11 months

1-2 years

3-4 years

≥5 years

10. **Do you use imaging to inform your management of PHP**? (i.e., ultrasound, MRI, X-ray)
    Likert Scale:
    Never
    Rarely
    Sometimes
    Frequently
    Very frequently

If yes, which imaging modalities do you use?

Likert scale

Magnetic resonance imaging (MRI)

Ultrasound

X-ray

If you use other imaging modalities please specify: (optional) 'Please do not enter any information that may identify you or anyone else in the box'.

11. **What imaging features do you feel are clinically important in plantar heel pain?**
    Likert
    Unsure
    Not important
    Slightly important
    Moderately important
    Important
    Very important

Bone marrow oedema

Heel fat pad thickness

Heel spurs

Inflammation

Plantar fascia thickness

Plantar fascia tears

If you feel other imaging features are important please specify: (optional) 'Please do not enter any information that may identify you or anyone else in the box'.

12. **What clinical factors do you consider important in your management of PHP?**

Please select importance (Likert)

Unsure
Not important
Slightly important
Moderately important
Important
Very important

Body mass index (BMI)

Co-morbidities

Clinical subgroups (i.e., muscle tightness, strength, range of motion)

Foot posture

Occupation (i.e. standing, active occupations)

Pain chronicity

Pain intensity

Pain type (i.e. nociceptive, neuropathic, nociplastic)

Psychosocial factors

Tissue subgroups (i.e., fasciopathy, inflammation, bone marrow oedema)

If you feel other clinical factors are important please specify: (optional) 'Please do not enter any information that may identify you or anyone else in the box'.

13. **Do you refer for cortico-steroid injections?**

List Likert Scale: Never, rarely, sometimes, frequently, very frequently

14. **Do you perform corticosteroid injections for plantar heel pain?**

List Likert Scale: Never, rarely, sometimes, frequently, very frequently

15. **Do you provide advice about medications to people with PHP?** Y/N

If Yes: What types of medication would you provide pharmacological advice on?

Please select all that apply

Non-steroidal anti-inflammatory drugs (NSAIDs)

Simple analgesics (e.g., paracetamol)

Other medications

If you selected, Other please specify: 'Please do not enter any information that may identify you or anyone else in the box'.

16. **Do you provide physical interventions/ treatments to people with PHP? Yes/No**

(This section is about interventions, treatments, or advice that you provide. Referrals to other services/ practitioners are covered in a later section.)

If Yes: How often do you provide the following physical interventions for people with PHP?

List Likert Scale: Never, rarely, sometimes, frequently, very frequently)

Acupuncture

Electrotherapy: ultrasound

Electrotherapy: shockwave therapy

Exercise: strengthening

Exercise: stretching

Exercise: balance/ proprioception

Manual therapy (joint or soft tissue)

Orthoses: Prefabricated/off the shelf

Orthoses: Custom made to a mould of their foot

Padding

Splinting

Taping/Strapping

17. As part of your PHP management what non-pharmacological advice/education do you routinely provide?

Please select all that apply:

Alcohol intake

Behavioural change

Footwear advice

Health education

Pacing/activity modification

Physical activity

Information about PHP (i.e. underlying condition/ pain mechanism/ prognosis)

Self-management

Smoking cessation

Weight-management

Other

If you selected, Other please specify: 'Please do not enter any information that may identify you or anyone else in the box'.

18. **What sources are most important in informing your management of PHP?**

Please select all that apply

Departmental guidelines

Clinical experience

Conferences

Published clinical guidelines

Research/ literature

Social media posts

Special interest groups

Other

If you selected, Other please specify: 'Please do not enter any information that may identify you or anyone else in the box'.

19. **Do you provide patient information resources as part of your management plan (i.e., exercise sheets, textual information, digital information)**

Yes/No

If yes, what type of patient information resources do you routinely provide?

Select all that apply

App based information

Paper information

Webpage information

Other

If you selected, Other please specify: 'Please do not enter any information that may identify you or anyone else in the box'.

20. **Do you have access to plantar heel pain patient information resources in languages other than English?**

Yes/No

Please could you specify in which other languages you have access to patient information resources? (Optional)

21. **Do you refer people with PHP on to other professions/practitioners for management?**

Please select all that apply

First Contact Practice

General Practice

Musculoskeletal (MSK) Service

Orthopaedic Service

Orthotics Service

Osteopathy Service

Physiotherapy Service

Podiatry Service

Rheumatology Service

Other

If you selected, Other please specify: 'Please do not enter any information that may identify you or anyone else in the box'.

Section 3

Outcome measurements

22. **What do you feel are the important outcomes in PHP?**

Please select all that apply

Activities of daily living

Adherence to the intervention/management plan

Function

Pain

Quality of life

Other

If you selected, Other please specify: 'Please do not enter any information that may identify you or anyone else in the box'.

23. **Do you measure patient reported outcomes as part of your PHP management?**

Likert Scale: (Never, rarely, sometimes, frequently, very frequently)

24. Do you use pain scales as part of your PHP management (i.e. VAS, NRS)?

Please select all that apply:

Numeric pain rating scale (NPRS) (i.e., 0-10)

Verbal rating scale (i.e., no pain, mild, moderate, severe)

Visual analogue scale (VAS) (i.e., 0-100 mm)

Other

If you selected, Other please specify 'Please do not enter any information that may identify you or anyone else in the box'.

What do you use the pain intensity scales to measure?

Select all that apply

Average pain

First step pain

Night pain

Other

If you selected, Other please specify: 'Please do not enter any information that may identify you or anyone else in the box'.

25. Do you use foot specific outcome measurement tools as part of your PHP management? Yes/No

If yes Please select all that apply

American Orthopaedic Foot and Ankle Society (AOFAS) Clinical Rating Scale

Foot and Ankle Ability Measure (FAAM)

Foot and Ankle Disability Index (FADI)

Foot and Ankle Outcome Score (FAOS)

Foot Function Index (FFI)

Foot Health Status Questionnaire (FHSQ)

Foot Posture Index (FPI)

Manchester Foot Pain and Disability Index (MFPDI)

Manchester-Oxford Foot Questionnaire MOXFQ

Self-reported Foot and Ankle Score (SEFAS)

Other

If you selected, Other please specify: 'Please do not enter any information that may identify you or anyone else in the box'.

26. **Do you use generic outcome measurement tools as part of your PHP management? (i.e., EQ5D, SF-36) Yes/No**

If yes, please select all that apply

EQ5D

Roles and Maudsley score

Short Form–36 (SF-36)

Other

If you selected, Other please specify: 'Please do not enter any information that may identify you or anyone else in the box'.

Section 4:

Service provision

27. **How many treatment sessions/consultations would you generally provide for someone with PHP in your service?**

List:

1-2

3-4

5-6

7-8

≥9

28. **Are there limits on the number of treatment/consultation sessions you can provide for people with PHP within your service?**
Yes/No/

If yes, what limits the number of treatment/consultation sessions you can provide to someone with PHP in your service?

Please tick all that apply

Service/commissioning agreements

Waiting list pressures

Staffing pressures

Other? Free text 'Please do not enter any information that may identify you or anyone else in the box'.

29. **Does your service have a waiting list to see people with PHP?**
    Yes/No

    Yes:
    On average, how long would someone with PHP have to wait to be treated in your service from point of referral? (please select one)

    <1 month
    1-2 months
    2-4 months
    4-6 months
    6-8 months
    8-10 months
    10-12 months
    ≥12 months

30. **What do you feel are the most important reasons for your service's current waiting list?**

Please select all that apply

High level of referrals

Staffing pressures

Covid related backlog

Other (free text) 'Please do not enter any information that may identify you or anyone else in the box'.

31. **Have you, or your service, had to limit care provision to people with PHP since the Covid-19 pandemic?**
    Yes/No/Unsure

Please could you provide more detail about your answer here. (optional) (free text) 'Please do not enter any information that may identify you or anyone else in the box.

Thank you for completing the survey! If you have any questions about the survey please contact the study's chief investigator at the University of Leeds, Chris Drake.

Email c.j.drake@leeds.ac.uk
