# Supplementary material for: Plantar Heel Pain Management: A Survey of UK Registered Healthcare Professionals
Source: J Foot Ankle Res. 2025 Oct 11;18(4):e70087. doi: 10.1002/jfa2.70087 (PMC12515048; doi:10.1002/jfa2.70087)
Supplement: Supplementary file 2 — Supporting Information S2 [file JFA2-18-e70087-s003.docx]

**Supplementary File 2.** Details of organisations distributing the online survey

| Organisation | Target Membership |
| --- | --- |
| Advanced Practice Physiotherapy Network (APPN) | Advanced Physiotherapy Practitioners |
| Association of Foot & Ankle Physiotherapists (AFAP) | Foot and ankle practitioners |
| British Association of Prosthetists and Orthotists (BAPO) | Orthotists |
| MSK:UK | Musculoskeletal podiatrists |
| Primary Care Rheumatology and Musculoskeletal Medicine Society (PRCMM) | GPs, nurses, and AHPs with a special interest in MSK and rheumatology |
| Social Media: Twitter/ X | Any healthcare professional involved in plantar heel pain management. |
| Facebook | UK Podiatry, Physiotherapy, Osteopathy. and other AHP groups. |
