# Supplementary material for: Plantar Heel Pain Management: A Survey of UK Registered Healthcare Professionals
Source: J Foot Ankle Res. 2025 Oct 11;18(4):e70087. doi: 10.1002/jfa2.70087 (PMC12515048; doi:10.1002/jfa2.70087)
Supplement: Supplementary file 3 — Supporting Information S3 [file JFA2-18-e70087-s002.docx]

**Supplementary File 3**

Free text comments were categorised and analysed descriptively as frequency (%).

**Question 10.** **What imaging modalities do you use?**

(n= 297)

**If you use other imaging modalities, please specify**

| Other modalities | Frequency (%) |
| --- | --- |
| Weightbearing CT | 3 (1) |

**Question 11.** **What imaging features do you feel are clinically important in plantar heel pain?**

(n=406)

**If you feel other imaging features are important, please specify**

| Other features | Frequency (%) |
| --- | --- |
| Bone features/pathology | 11 (2.7) |
| Plantar fat pad features | 10 (2.5) |
| Rheumatological features | 7 (1.7) |
| Muscle features | 6 (1.5) |
| Other plantar fascia features | 5 (1.2) |
| Nerve features | 3 (0.7) |
| Tumour | 3 (0.7) |

**Question 12. What clinical factors do you consider important in your management of PHP?**

(n= 406)

**If you feel other clinical factors are important, please specify**

| Other clinical factors | Frequency (%) |
| --- | --- |
| Footwear | 19 (4.7) |
| Activity levels | 16 (3.9) |
| Biomechanics | 12 (3) |
| Trauma | 9 (2.2) |
| Patient demographics | 9 (2.2) |
| Hormonal influences | 5 (1.2) |

**Question 15.** **What types of medication would you provide pharmacological advice on?**

n= (272)

**If you selected ‘Other’, please specify**

| Other medication advice | Frequency (%) |
| --- | --- |
| Neuropathic medications | 7 (2.6) |
| Co-codamol | 2 (0.7) |
| Disease modifying drugs | 2 (0.7) |
| Steroids | 1 (0.4) |

**Question 21.** **Do you refer people with PHP on to other professions/practitioners for management?**

n= (321)

**If you selected ‘Other’, please specify:**

| Other referrals | Frequency (%) |
| --- | --- |
| Pain services | 5 (1.6) |
| Radiology | 4 (1.2) |
| Sports medicine | 3 (0.9) |

**24. What do you use pain intensity scales to measure?**

n= (323)

**If you selected, Other please specify:**

| Other pain scale | Frequency (%) |
| --- | --- |
| Best and worst pain | 31 (9.6) |

**26.Do you use generic outcome measurement tools as part of your PHP management?**

n= (75)

**If you selected, Other please specify:**

| Other outcome tools | Frequency (%) |
| --- | --- |
| MSK HQ | 13 (17.3) |
| PSFS | 3 (4) |
| TOMS | 2 (2.7) |

**28. Are there limits on the number of treatment/consultation sessions you can provide for people with PHP within your service?**

n= (80)

**If you selected, Other please specify:**

| Other limitations | Frequency (%) |
| --- | --- |
| First point of contact service | 8 (10) |

**30. What are the most important reasons for your service’s current waiting list?**

n= (193)

**If you selected, Other please specify:**

| Other reasons | Frequency (%) |
| --- | --- |
| Clinic room availability | 2 (1) |

**31. Have you, or your service, had to limit care provision to people with PHP since the Covid-19 pandemic?**

n= (406)

**Please could you provide more detail about your answer:**

| Care limitations | Frequency (%) |
| --- | --- |
| Altered care provision | 13 (3.2) |
| Non-urgent care limits | 5 1.2) |
